# Prophylactic Nailing of Incomplete Atypical Femoral Fractures

**DOI:** 10.1155/2013/450148

**Published:** 2013-01-09

**Authors:** Chang-Wug Oh, Jong-Keon Oh, Ki-Chul Park, Joon-Woo Kim, Yong-Cheol Yoon

**Affiliations:** ^1^Department of Orthopedic Surgery, Kyungpook National University Hospital, 50, 2-Ga, Samdok, Chung-gu, Daegu 700-721, Republic of Korea; ^2^Department of Orthopedic Surgery, Korea University Guro Hospital, 148, Guro-dong, Guro-gu, Seoul 152-703, Republic of Korea; ^3^Department of Orthopedic Surgery, Hanyang University Guri Hospital, 153, Gyeongchun-ro, Guri, Gyeonggi-do 471-701, Republic of Korea

## Abstract

*Introduction*. Recent reports have described the occurrence of low-energy subtrochanteric and femoral shaft fractures associated with long-term bisphosphonate use. Although information regarding the surgical treatment of these atypical femoral fractures is increasing, it is unclear if the preventive operation is useful in incomplete fractures. This study examined the results of preventive intramedullary nailing for incomplete atypical femoral fractures. *Material and Methods*. A retrospective search was conducted for patients older than 50 years receiving bisphosphonate therapy, with incomplete, nondisplaced fractures in either the subtrochanteric or diaphyseal area of the femur. Seventeen patients with a total of 20 incomplete, non-displaced lesions were included. The mean duration of bisphosphonate use was 50.5 months. Eleven of the 17 (64.7%) patients had complete or incomplete fractures on the contralateral femur. All were treated with prophylactic fixation of an intramedullary (IM) nail. The minimum followup was 12 months. *Results*. All cases healed with a mean period of 14.3 weeks. Nineteen of the 20 cases healed with the dissolution of incomplete fractures of the lateral aspect. A complete fracture developed at the time of nailing in one patient, but it healed with callus bridging. *Conclusion*. IM nailing appears to be a reliable way of preventing the progress of incomplete atypical femoral fractures.

## 1. Introduction

Bisphosphonate (BP) medication has been the mainstay treatment of postmenopausal osteoporosis and metastatic diseases to the skeleton [1–4]. Although its efficacy is well known, several reports have described the bisphosphonate-associated insufficiency fractures of the femur among patients associated with long-term BP use [5]. Some studies have suggested that chronic suppression of bone turnover may produce hypermineralized bone, which is more brittle. These fractures differ from the typical proximal femoral fracture associated with osteoporosis. They are caused by low-energy mechanisms, with the typical radiographic features of unicortical beaking and hypertrophy of the diaphyseal cortex, appearing as insufficiency fractures [6, 7]. The prefracture radiographs of atypical femoral fractures have been described and include cortical thickening or beaking as well as a transverse line in the femoral cortex [8]. Several studies have reported that MR may find an incomplete lesion of atypical femoral fractures. These lesions have the potential to progress to complete fractures with associated thigh pain.

The decision to treat incomplete atypical femoral fractures nonsurgically or surgically is controversial. Although activity modification with partial weight bearing on the affected extremity is an option, it does not appear to be a reliable way of treating these fractures because the majority progress to fracture completion [9, 10]. Prophylactic fixation may prevent the fracture from progressing and the related morbidity. On the other hand, few studies have reported the results and risks of prophylactic fixation [11]. This study describes the ultimate outcomes of patients with incomplete atypical femoral fractures treated with intramedullary nailing.

## 2. Patients and Methods

A search of the fracture databases from three trauma centers was performed to identify patients older than 50 years with incomplete, non-displaced stress fractures in either the subtrochanteric or diaphyseal area of the femur between January 2008 and August 2010. Of these, 17 patients (20 fractures) with fractures radiographically characteristic of a bisphosphonate-associated incomplete stress femoral fracture were included in this study. The study design and protocol were approved by the Institutional Review Board. The inclusion criteria were as follows: (1) incomplete atypical femur fracture, as defined on the radiographs, (2) prophylactic IM nailing for incomplete fractures, and (3) clinical followup for at least one year after the index operation.

Eleven of the 17 (64.7%) patients (14 fractures) had complete or incomplete fractures of the contralateral femur. The other 6 patients did not show these lesions up to the latest followup. All except for three had a documented history of bisphosphonate use at the time of presentation. All patients were female with a mean age of 68.3 years (range, 54~83). The bone mineral density was evaluated in all cases by dual-energy X-ray absorptiometry (DEXA), which showed a mean *T*-score of −2.97 (range, −1.7~−5.2). Eleven of the 14 patients (78.6%) had been treated with alendronate, two patients were treated with risedronate, and one patient was treated with ibandronate. The average length of treatment with bisphosphonates was 50.5 months (range, 6~102). All patients had a minimum followup of 12 months (mean, 20.1; range, 12~33). All patients were recalled specifically for this study to assess the current physical status. The data was also obtained from medical records and radiographs.

**Figure 1:**
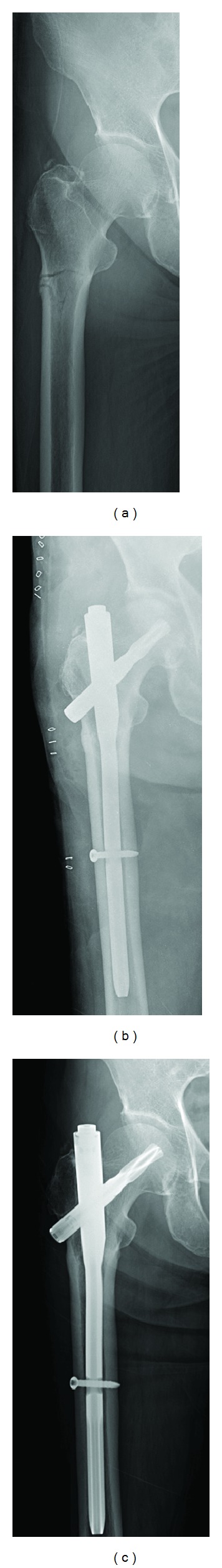
(a) A 72-year-old woman had pain in her right thigh with a transverse fracture line and thickening of the lateral cortex in the subtrochanteric area. (b) She underwent internal fixation using a proximal femoral nail. (c) At 13 weeks postoperatively, she had no pain with a dissolution of the fracture line at the lateral cortex.

Six fractures were located in the subtrochanteric area and 14 were located in the femoral shaft. All except one fracture lines located within lateral cortex only, whereas one fracture at subtrochanteric region involved more than 2/3 cortical width without displacement (Figure 1). All patients were treated with prophylactic fixation of an intramedullary nail. Eleven patients with incomplete contralateral femoral fractures were also nailed simultaneously or after primary operation. Closed IM nailing with static mode was performed in all cases. The entries of the nail were piriformis fossa in 3 patients and the tip of the greater trochanter in 17 cases. In the types of IM nail, a standard interlocking nail was used in 9 cases, and a cephalomedullary or reconstruction type nail was used in 11 cases.

All patients began hip and knee motion exercises, and weight bearing as tolerated was allowed immediately after surgery. Routine follow-up radiographs were obtained every 6~8 weeks until the fracture line vanished. Radiographic healing was documented as a loss of fracture lucency on the standard anteroposterior and lateral femoral radiographs taken at the standard follow-up intervals, whereas clinical healing was documented as an absence of pain (in those who presented without fracture lucency) and/or a loss of fracture lucency on the radiographs.

## 3. Results

All cases healed with a mean period of 14.3 weeks (Figure 1). None of the patients showed any limited motion of the hip and knee joints, and they could perform their normal daily activities. All could walk without crutches after a mean of 6 months (4~8 months).

**Figure 2:**
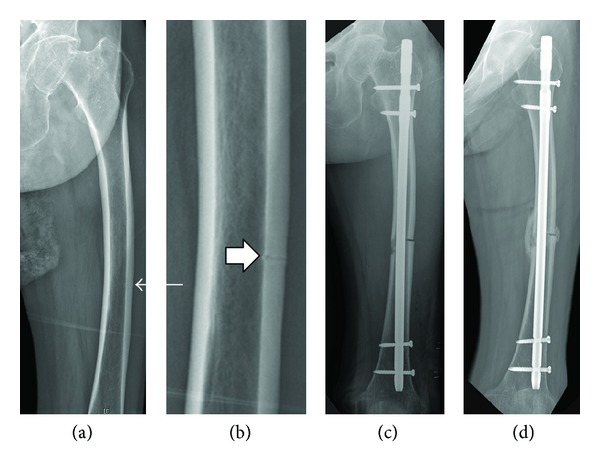
(a) A 74-year-old woman suffered thigh pain and her AP radiograph showed exaggerated femoral bowing with a transverse radiolucent line (arrow) in the lateral cortex of the distal 1/3 of the left femur. (b) A magnified view (box arrow) of the lesion revealed an incomplete, prefracture lesion of an atypical femoral fracture. (c) After preventive IM nailing, a complete fracture occurred. (d) The union was achieved with callus bridging at 18 weeks postoperatively.

Nineteen of the 20 cases healed with the dissolution of the incomplete fracture of the lateral aspect. A complete fracture developed at the time of nailing in one patient, but it healed with callus bridging by 18 weeks after surgery. This complication was attributed to a mismatch of curvature between the femoral bow and IM nail (Figure 2). 

With the exception of 3 patients with a complete atypical femoral fracture on the contralateral side, which were operated simultaneously, the average hospital stay was 5.8 days. 

No patients had hardware removed for symptomatic reasons after the completion of fracture healing. No infections were documented in any of the study patients.

## 4. Discussion

Over the past few years, a number of case series have suggested an association between low-energy atypical fractures of the femur and BP use for osteoporosis management, even though some of non-BP users also developed similar lesions [12]. The definition, incidence, and characteristics of atypical femoral fractures are unclear, because of multiple associated risk factors [13]. But, a recent nation-wide study showed a high prevalence of current bisphosphonate use among patients with atypical fractures and its relative risk about 47 in the cohort analysis [14]. Therefore, for patients receiving BP therapy and who reported the symptoms of pain originating from the femur, an appropriate radiographic examination of both femurs is recommended to find any suspicious lesions, including the prefracture radiographic findings. Several studies [9, 10] reported that the spontaneous healing of atypical femur fractures was not expected. Most non-displaced fractures progressed to fractures with secondary displacement, and complete fractures inevitably developed even with a low-energy injury. Although established atypical femoral fractures require surgical treatment, there are few reports of surgical treatment to prevent incomplete lesions from progressing to complete fractures. In this study, 20 cases of incomplete or non-displaced lesions were found. The aim of this study was to determine the efficacy and results of preventive IM nailing in patients with incomplete lesions of atypical femoral fractures.

The union rate varies after the surgical treatment of complete atypical femoral fractures. Although Capeci and Tejwani [6] reported that all 7 fractures achieved union after reamed IM nailing, they included four cases of non-displaced lesions. On the other hand, in a recent study excluding the prefracture lesions [15], the healing of atypical femoral fractures after IM nailing was unsatisfactory with a low union rate (54%), and many patients required additional procedures. The result of 100% union means that prophylactic IM nailing is a meaningful method. This is in contrast to the long duration of healing and the late return to normal daily activities after non-surgical treatment, which is successful only in a small proportion of cases.

Traditionally, IM nailing of femoral shaft fractures was reported to be a very successful surgical procedure, with a 98% to 99% healing rate and a very low complication rate [16]. On the other hand, Weil et al. [15] reported a higher failure rate of IM nailing in atypical femoral fractures. They insisted that these fractures might reflect an impaired bone healing process rather than the differences in surgical technique. In this series, there was only one minor complication of further fracture during nail insertion, which healed without secondary procedures. This is comparable to the report of a high complication rate in a series of complete fractures [17]. IM nailing is easier before than after fracture completion, and the healing time is much less. In addition, there is a shorter postoperative hospital stay. Therefore, the significance of preventive nailing is noteworthy.

The most frequent complication of IM nailing of atypical femoral fractures was intraoperative femoral shaft comminution during nail insertion up to 29.4% [17]. This complication was also experienced in non-displaced femoral shaft fractures in the present study, which appears to be an iatrogenic fracture during nail placement. Lim et al. [18] reported that femoral bowing deformities are a high-risk factor of femoral insufficiency fractures. The mismatch of curvature between the implant and femur is believed to be the reason for this intraoperative complication due to the observation of exaggerated femoral bowing in the present case.

This study had several limitations. This study was a retrospective case series, and the sample size of this study was relatively small. Because of the different locations of pathologic lesions, the nails used were not the same. In addition, plate fixation was not included as a preventive operation, even though IM nails can be impractical in certain situations. In a recent report [15], the results of plate fixation were unsatisfactory in atypical femoral fractures and they were excluded from the study design. Therefore, a prospective randomized trial with a larger cohort will be needed to compare IM nailing with plate fixation.

## 5. Conclusion

Prophylactic fixation of atypical femoral fractures is recommended. Despite the small number of patients who underwent prophylactic fixation, this procedure appears to achieve a more efficient postoperative course.
